# BCA2/Rabring7 Promotes Tetherin-Dependent HIV-1 Restriction

**DOI:** 10.1371/journal.ppat.1000700

**Published:** 2009-12-18

**Authors:** Kei Miyakawa, Akihide Ryo, Tsutomu Murakami, Kenji Ohba, Shoji Yamaoka, Mitsunori Fukuda, John Guatelli, Naoki Yamamoto

**Affiliations:** 1 AIDS Research Center, National Institute of Infectious Diseases, Shinjuku-ku, Tokyo, Japan; 2 Department of Molecular Virology, Graduate School of Medicine, Tokyo Medical and Dental University, Bunkyo-ku, Tokyo, Japan; 3 Department of Developmental Biology and Neurosciences, Graduate School of Life Sciences, Tohoku University, Sendai, Miyagi, Japan; 4 Department of Medicine, University of California San Diego, La Jolla, California, United States of America; University of Geneva, Switzerland

## Abstract

Host cell factors can either positively or negatively regulate the assembly and egress of HIV-1 particles from infected cells. Recent reports have identified a previously uncharacterized transmembrane protein, tetherin/CD317/BST-2, as a crucial host restriction factor that acts during a late budding step in HIV-1 replication by inhibiting viral particle release. Although tetherin has been shown to promote the retention of nascent viral particles on the host cell surface, the precise molecular mechanisms that occur during and after these tethering events remain largely unknown. We here report that a RING-type E3 ubiquitin ligase, BCA2 (Breast cancer-associated gene 2; also called Rabring7, ZNF364 or RNF115), is a novel tetherin-interacting host protein that facilitates the restriction of HIV-1 particle production in tetherin-positive cells. The expression of human BCA2 in “tetherin-positive” HeLa, but not in “tetherin-negative” HOS cells, resulted in a strong restriction of HIV-1 particle production. Upon the expression of tetherin in HOS cells, BCA2 was capable of inhibiting viral particle production as in HeLa cells. The targeted depletion of endogenous BCA2 by RNA interference (RNAi) in HeLa cells reduced the intracellular accumulation of viral particles, which were nevertheless retained on the plasma membrane. BCA2 was also found to facilitate the internalization of HIV-1 virions into CD63^+^ intracellular vesicles leading to their lysosomal degradation. These results indicate that BCA2 accelerates the internalization and degradation of viral particles following their tethering to the cell surface and is a co-factor or enhancer for the tetherin-dependent restriction of HIV-1 release from infected cells.

## Author Summary

Human cells possess multiple systems that render them resistant to viral infection. Recently, a transmembrane protein, tetherin, has been identified as an antiviral host factor in HIV-1-infected cells. Tetherin retains newly assembled virions at the plasma membrane and prevents viral release from the infected cells. However, the precise molecular mechanisms following the virion tethering remain largely unknown. In our current study, we have identified a RING-type E3 ubiquitin ligase, BCA2, which co-localizes and interacts with tetherin in human cells. BCA2 was found to facilitate the internalization of HIV-1 particles captured by tetherin on the plasma membrane and to enhance the targeting of viral particles to the lysosomes. Conversely, the targeted depletion of endogenous BCA2 reduces the intracellular accumulation of viral particles. Additionally, the expression of a small viral protein Vpu, an antagonist of tetherin, counteracts the antiviral effects of BCA2. These results suggest that BCA2 is a potential antiviral factor that collaborates with tetherin to facilitate the degradation of nascent HIV-1 particles during “post-tethering” processes.

## Introduction

The human immunodeficiency virus (HIV) exploits the host cell machinery to maximize viral particle production [1]. In contrast, there are multiple systems in host cells that render them resistant to viral infection through the actions of innate host cell restriction factors [2],[3]. This intracellular innate system can in turn be antagonized by certain viral proteins, creating a conflict between host cells and pathogens. There is accumulating evidence to now suggest that the balance between host and viral factors influences the susceptibility of the host cells to HIV infection and ultimately AIDS progression [4].

A human transmembrane protein, tetherin (also known as BST-2, CD317 or HM1.24) has been identified as an interferon-induced antiviral host factor in HIV-1-infected cells. During the late phase of the viral replication pathway, tetherin retains nascent HIV-1 virions at the plasma membrane and prevents viral spread [5]–[7]. Tetherin has been shown not only to block the release of lentiviruses such as HIV-1 or SIV, but also other viruses such as MLV, HTLV-1, Lassa virus and the Marburg virus [8]–[10]. These results indicate that tetherin has broad antiviral properties through the inhibition of viral particle release, and therefore that the activation of this protein might be an effective strategy as an anti-viral therapy.

Viral Protein U (Vpu) is a 16 kD phosphoprotein that is encoded almost exclusively by SIV_CPZ_ and its descendants, including HIV-1 [11]–[13]. Vpu is a factor that facilitates viral particle release by antagonizing tetherin-mediated viral restriction [6],[7],[14],[15], in addition to its effects upon CD4 degradation [16]–[18]. The expression of Vpu has been shown to downregulate the tetherin levels on the plasma membrane resulting in effective virion release [7],[19]. Indeed, Vpu-defective HIV-1 virions are efficiently retained on the plasma membrane and fewer viral particles are released compared with wild-type virions in tetherin-positive cells, including T cells and macrophages. [14],[20]. On the other hand, in tetherin-negative cells, viral particle release is much less affected by either the presence or absence of Vpu [6],[7]. These results suggest that Vpu antagonizes the function of tetherin, which otherwise restricts the release of HIV-1 from infected host cells. Following cell surface tethering, HIV-1 virions are subjected to internalization into CD63-positive endosomal compartments, thereby limiting the extent of virus spread [6], [15], [21]–[25]. Although tetherin can hold nascent viral particles on the cell surface of the host cells, the precise molecular events following the virion tethering and identity of the related host factors that regulate these processes remain largely unknown.

In our current study, we identify a RING-type E3 ubiquitin ligase, BCA2 (breast cancer associated gene 2; identical to Rabring7, ZNF364 or RNF115) as a novel tetherin-interacting protein that enhances tetherin-dependent viral restriction. BCA2 was found to facilitate the internalization of HIV-1 particles captured by tetherin on the plasma membrane by associating with the cytoplasmic tail of tetherin and directing the degradation of viral particles in lysosomes. Significantly, the targeted depletion of BCA2 was found to reduce the intracellular accumulation of viral particles and to increase the persistence of nascent virions on the plasma membrane. Our current results thus reveal that BCA2 is a potential antiviral host factor through its collaboration with tetherin and is therefore a potential new therapeutic target for AIDS and its related disorders.

## Results

### Identification of BCA2 as a tetherin-interacting protein

**Figure 1:**
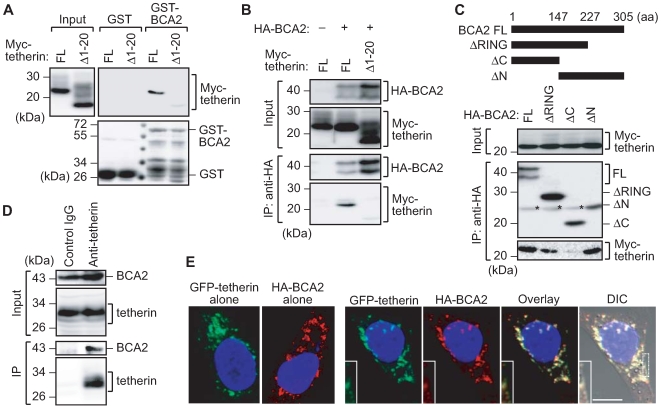
BCA2 is a tetherin-interacting protein. (A) GST pull-down analysis of 293T cells expressing either N-terminally Myc-tagged-tetherin (FL) or a mutant lacking the cytoplasmic tail domain (Δ1–20). Cell lysates were precipitated with either purified GST or GST-BCA2, followed by immunoblotting analysis with a Myc antibody to detect BCA2 binding (top panels). To control for the expression levels of GST, a Coomassie Brilliant Blue stained image is also shown (bottom panel). (B) Immunoprecipitation analysis of 293T cells expressing N-terminally HA-tagged-BCA2 together with either Myc-tetherin (FL) or its mutant (Δ1–20). Cell lysates were immunoprecipitated with HA antibodies, followed by immunoblotting analysis with either HA or Myc antibodies. (C) Immunoprecipitation analysis of 293T cells expressing Myc-tetherin together with HA-BCA2 (FL) or its deletion mutants (ΔRING, ΔC and ΔN). Asterisks indicate non-specific IgG bands. (D) Immunoprecipitation analysis of endogenous tetherin and BCA2. HeLa cell lysates were immunoprecipitated with either anti-tetherin monoclonal antibody or control mouse IgG followed by immunoblotting with the indicated antibodies. (E) Confocal microscopic analysis of HeLa cells expressing GFP-tagged tetherin and HA-BCA2 (scale bar, 10 µm). Cells were fixed, permeabilized and stained with HA antibodies (red) followed by confocal microscopy. The inset shows an expanded view of the area indicated by the white box in which an association of GFP-tetherin with HA-BCA2 at the plasma membrane is evident.

The precise mechanism in which HIV-1 particles undergo internalization and/or degradation in cells following tetherin-mediated capture on the plasma membrane has not been well characterized. However, accumulating evidence now suggests that plasma membrane-tethered virions transit the small G protein Rab-dependent endocytotic pathway [6],[15]. To delineate the molecular determinants that regulate this process, we attempted to identify the Rab family member or its effector proteins that functionally interact with tetherin. As our initial screening test, we performed immunoprecipitation and GST-pull down analyses to examine the interaction of approximately 60 Rab family proteins with either tetherin or HIV-1 Gag protein. These in vitro interaction assays revealed that a Rab7-interacting protein, BCA2, could interact with tetherin (data not shown). To further confirm this interaction, we performed GST-pull down analysis with recombinant GST-BCA2. 293T cells were transfected with either N-terminal Myc-epitope-tagged wild-type tetherin or its deletion mutant devoid of the cytoplasmic tail domain (tetherinΔ1–20). Cell lysates were then subjected to GST-pull down analysis with either GST alone or GST-BCA2. Consequently, GST-BCA2 was found to interact with full-length tetherin in cell lysates, but to interact less efficiently with tetherinΔ1–20 (Fig. 1A). This result was further confirmed by immunoprecipitation analysis using 293T cells transfected with either Myc-tetherin or Myc-tetherinΔ1–20 together with an N-terminal HA-tagged BCA2 construct (Fig. 1B).

BCA2 contains an N-terminal Rab7 binding domain and a C-terminal RING domain [26]. To investigate which of these is involved in the interaction with tetherin, we constructed BCA2 derivatives lacking these domains for use in immunoprecipitation analysis. Our results demonstrated that Myc-tetherin is efficiently coimmunoprecipitated with the full length BCA2, the N-terminal truncation mutant, BCA2ΔN (148–305 aa) or the RING domain deleted mutant, BCA2ΔRING (1–227 aa) (Fig. 1C). However, the C-terminal truncation mutant, BCA2ΔC (1–147 aa), showed no detectable interaction with Myc-tetherin (Fig. 1C). These results suggest that tetherin can interact with the middle portion of BCA2 (147–227 aa) located between the Rab-interacting domain and RING finger domain. We also confirmed an interaction between endogenous BCA2 and tetherin in HeLa cells (Fig. 1D), where these proteins were verified to be endogenously expressed (Fig. S1).

To further verify the association between BCA2 and tetherin in cells, we examined the intracellular localization of these two proteins using confocal microscopy. Immunofluorescent analysis revealed that N-terminal GFP-tagged tetherin and HA-BCA2 show a similar distribution in cells and form multiple cytoplasmic dots when they are expressed alone (Fig. 1E). When GFP-tetherin and HA-BCA2 are co-transfected however, these proteins show a significant co-localization predominantly in the cytoplasm, but also in part at the plasma membrane (Fig. 1E). These results together indicate that BCA2 is a tetherin-interacting protein that associates with the cytoplasmic tail of tetherin in cells.

### BCA2 facilitates the restriction of HIV-1 particle production in cells expressing tetherin

**Figure 2:**
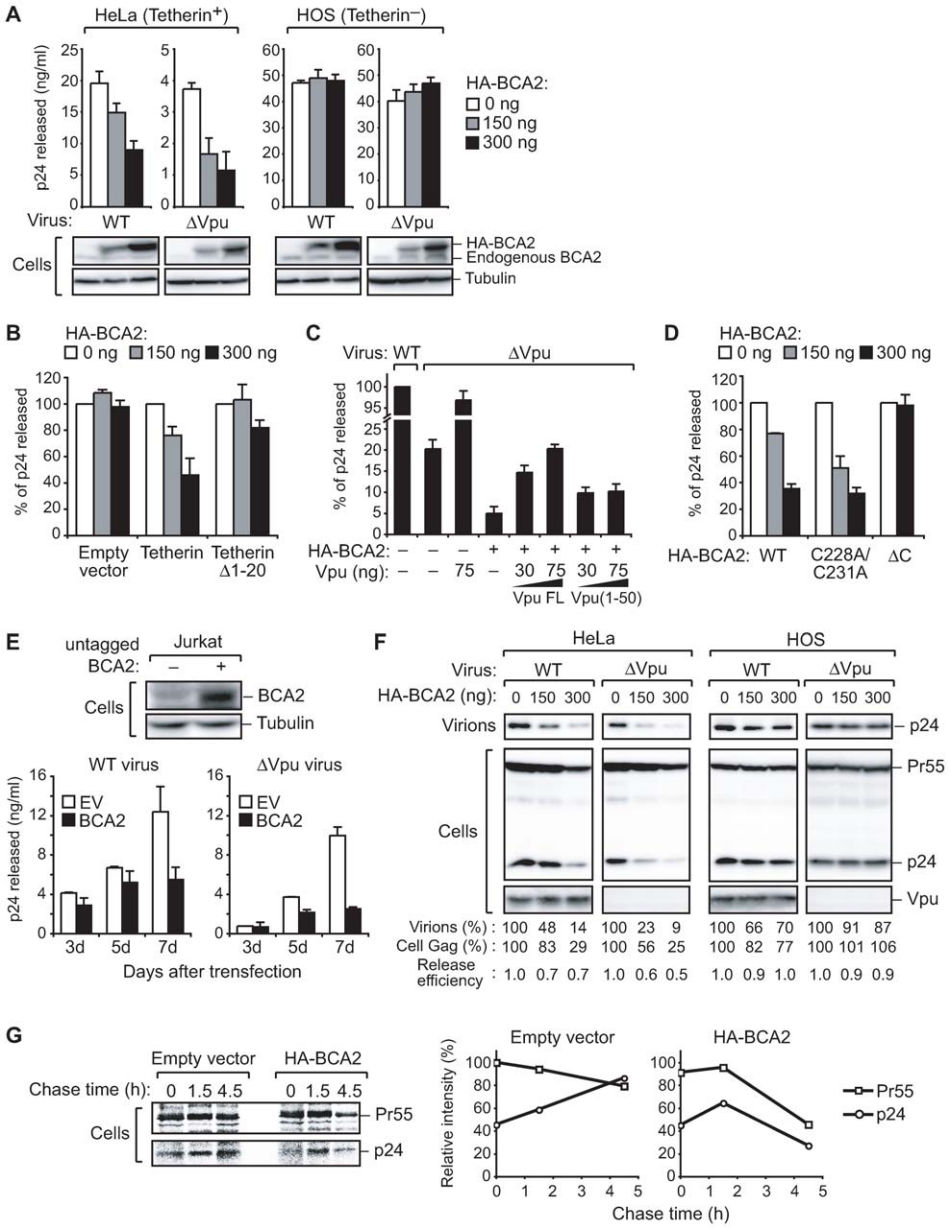
BCA2 inhibits HIV-1 particle production in cells expressing tetherin. (A) Single-round virus release analysis was performed using the indicated cell types transfected with either 300 ng of pNL4–3 or pNL4–3ΔVpu along with the indicated amounts of pCMV-HA-BCA2. At 48 hours following transfection, cell supernatants were analyzed by p24 ELISA. Immunoblotting with a BCA2 antibody for both endogenous and HA-tagged BCA2 expression is shown in the bottom panels. (B) Tetherin-dependent effects of BCA2 on HIV-1 particle production. HOS cells were transiently transfected with 100 ng of pCMV-Myc-tetherin or its deletion mutant (Δ1–20, lacking the cytoplasmic tail) together with 300 ng of pNL4–3 and indicated amounts of pCMV-HA-BCA2, followed by p24 ELISA. (C) Vpu antagonizes the effects of BCA2 upon HIV-1 particle production. HeLa cells were transiently transfected with either the indicated amounts of Vpu or its deletion mutant (1–50, lacking a portion of the cytoplasmic domain) and 300 ng of pNL4–3ΔVpu was co-transfected with or without 300 ng of pCMV-HA-BCA2. After 48 hours, cell supernatants were analyzed by p24 ELISA. (D) Single-round virus release analysis was performed using HeLa cell transfected with 300 ng of pNL4–3 along with either pCMV-HA-BCA2 (WT), its RING finger-defective mutant (C228A/C231A) or tetherin-interacting motif defective BCA2 mutant (ΔC). At 48 hours following transfection, viral supernatants were analyzed by p24 ELISA. (E) Jurkat cells were transfected with either empty vector (EV) or pIRESpuro-BCA2 by electroporation and selected with puromycin for 24 hours. The stable expression of BCA2 on Jurkat cells was confirmed by BCA2 immunoblotting (top panel). Cells were then infected with either HIV-1_NL4–3_ or HIV-1_NL4–3_Δ_Vpu_ at a low multiplicity. Cell supernatants were harvested at the indicated time-points and subjected to p24 ELISA (bottom panel). (F) BCA2 reduces the level of cell-associated Gag protein. Immunoblotting analysis of the cell lysates described in (A) was performed. The numerical values below the blots indicate the Gag signal intensities determined by densitometry. The virus release efficiency was calculated as “Sup Gag per Total Gag (Cell Gag plus Sup Gag)”. (G) Pulse-chase analysis of HeLa cells transfected with pNL4–3ΔVpu together with either control vector or pCMV-HA-BCA2. Two days after transfection, the radiolabeled cells were harvested at the indicated times, and cell lysates were immunoprecipitated with anti-p24 antibody, and then analyzed by SDS-PAGE and autoradiography (left panel). The relative intensity of Gag bands was determined by densitometry (right panel).

We next examined the effects of BCA2 upon HIV-1 particle production in both endogenously tetherin-positive HeLa cells and in tetherin-negative HOS cells. Endogenous tetherin expression on the cell surface was confirmed by flow cytometric analysis (Fig. S1A). Cells were transfected with different amounts of HA-BCA2 together with either the HIV-1 proviral plasmid (pNL4–3) [27] or a Vpu-deleted version of this construct (pNL4–3ΔVpu) [14]. After 48 hours, cell supernatants were assayed for the Gag p24 antigen. Strikingly, the expression of BCA2 in tetherin-positive HeLa cells led to a strong restriction of HIV-1 particle production. Importantly, the restriction of Vpu-deleted HIV-1 was more prominent than that of the WT virus in HeLa cells (Fig. 2A). However, there was no significant suppressive effect of BCA2 on viral particle production in tetherin-negative HOS cells (Fig. 2A). This indicated that BCA2 reduces HIV-1 particle production in the presence of tetherin. Consistent with this observation, HOS cells exogenously expressing relatively low amounts of tetherin, but not the tetherinΔ1–20 mutant, showed BCA2-mediated restriction of HIV-1 particle production (Fig. 2B).

Since Vpu has been shown to antagonize the antiviral activity of tetherin [6],[7], we next investigated whether Vpu could also counteract the antiviral effects of BCA2. As expected, Vpu-defective HIV-1 particle production was almost completely recovered by the expression of Vpu in HeLa cells (Fig. 2C). However, the co-expression of BCA2 significantly suppressed the recovery of virus particle production by Vpu (Fig. 2C). Conversely, the expression of a truncated Vpu mutant (Vpu1–50), the function of which is partly impaired [6], only partially counteracted HIV-1 restriction by BCA2 (Fig. 2C). These results indicate that a functional Vpu antagonizes the restrictive activity of BCA2. Together with our finding that BCA2 can restrict HIV-1 particle production only in tetherin-expressing cells, these data indicate that the function of tetherin, which is antagonized by Vpu, is likely required for the BCA2-mediated restriction of HIV-1 particle production.

Previous studies have demonstrated that BCA2 has E3 ubiquitin ligase activity which is essential for the downregulation of EGFR expression [28]. We therefore examined whether this activity is necessary for the anti-viral effects of BCA2. We created a RING finger-defective mutant BCA2 (C228A/C231A) [29] and investigated its effect upon virus particle production. Although a tetherin-interacting motif defective BCA2 mutant (BCA2ΔC) failed to restrict viral particle production, both WT and C228A/C231A BCA2 were capable of doing so (Fig. 2D). Moreover, the effect of C228A/C231A BCA2 mutant was modest increase than that of WT BCA2 (Fig. 2D), probably due to the inhibition of both auto-ubiquitination and following degradation of this mutant as reported previously [28]. These results indicate that the ubiquitin ligase activity of BCA2 is dispensable for its function in the restriction of virus particle formation.

To next investigate the effects of BCA2 upon virus particle restriction in T cells, we created Jurkat cells stably expressing untagged BCA2 (Fig. 2E). FACS analysis with a tetherin antibody revealed that these cells indeed express tetherin on their cell surface (Fig. S1A). The cells were then infected with either HIV-1_NL4–3_ or HIV-1_NL4–3_Δ_Vpu_ at a low multiplicity of infection (m.o.i. = 0.05). In agreement with a previous report [11], we found that Vpu-deleted virus replicated slightly less efficiently than WT-virus (Fig. 2E). Our results showed that BCA2 expression reduces HIV-1 particle production in both WT- and ΔVpu-virus infected cells, although this effect was more prominent in cells infected with ΔVpu-virus (about 4-fold) than with WT-virus (about 2-fold) (Fig. 2E). Interestingly, immunoblotting analysis revealed that the expression levels of endogenous BCA2 in HeLa and Jurkat cells were relatively lower than in HOS cells (Fig. S1B), implying that exogenous BCA2 expression would tend to impact virus particle restriction in these cells in the presence of functional tetherin.

To delineate the molecular mechanism by which BCA2 suppresses virus production, we performed immunoblotting analysis with a p24 antibody. Interestingly, the expression of BCA2 in HeLa cells significantly reduced the Gag protein levels, particularly cell-associated p24, but had no effect upon the expression of Vpu (Fig. 2F). Our results also indicate that BCA2 expression has modest effects on viral release efficiency as compared with its drastic effects on the cell-associated p24 protein levels (Fig. 2F). Of note, the BCA2-induced depletion of cell-associated p24 in the absence of Vpu was more prominent than in the presence of Vpu (Fig. 2F). These data together suggest that BCA2 may enhance the degradation of nascent HIV-1 virions captured by tetherin on the plasma membrane.

To rule out the possibility that BCA2 affects the expression of HIV-1 proteins, we next performed pulse-chase analysis with pNL4-3ΔVpu-transfected HeLa cells. Our results demonstrated that BCA2 expression induces the rapid degradation of the HIV-1 Gag protein (Fig. 2G). Consistent with our immunoblotting data (Fig. 2F), the degradation of p24 was shown to be more prominent than that of Pr55 (Fig. 2G). Furthermore, our RT-PCR analysis revealed that BCA2 expression does not significantly affect the mRNA levels of HIV-1 Gag (Fig. S2). These results together indicate that BCA2 facilitates the intracellular degradation of virus particles rather than the suppression of HIV-1 protein expression.

### BCA2 promotes the accumulation of HIV-1 virions in intracellular compartments

**Figure 3:**
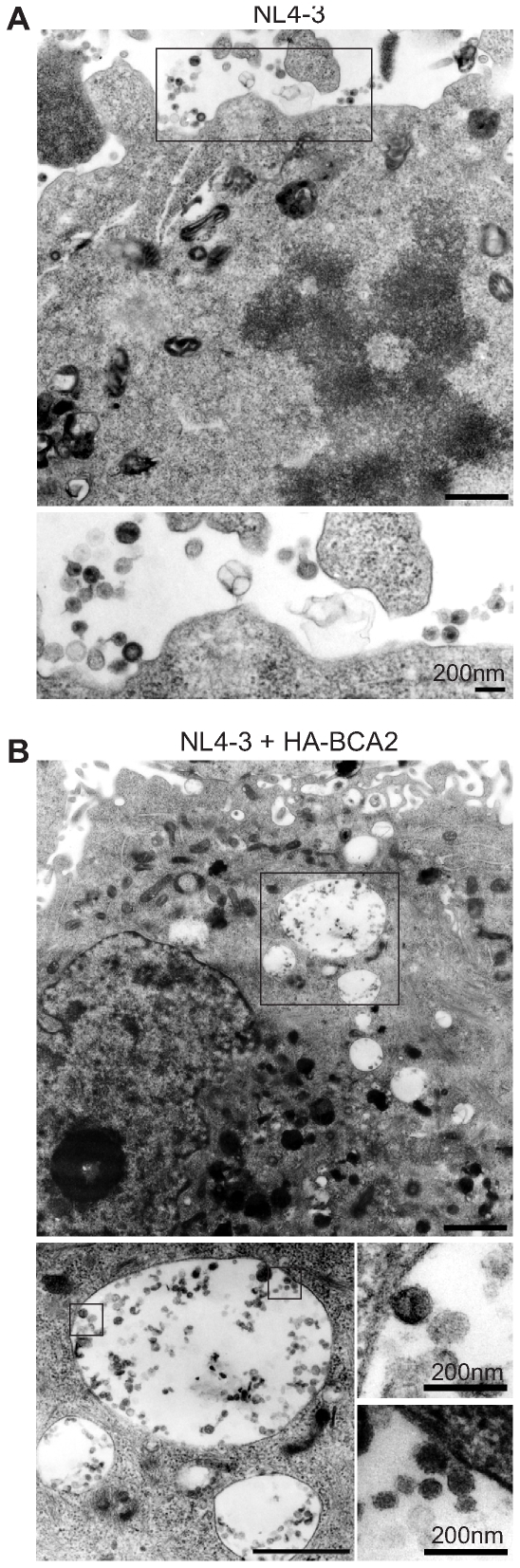
BCA2 promotes the accumulation of HIV-1 virions in intracellular compartments. Electron microscopic analysis of HeLa cells transfected with pNL4–3 and either control vector (A) or pCMV-HA-BCA2 (B), at a molar ratio of 1∶3. (Scale bars, 1 µm except where indicated).

As described above, the expression of BCA2 significantly reduces the level of cell-associated p24 protein, raising the possibility that it facilitates the intracellular degradation of unreleased virions. To test this possibility, we performed transmission electron microscopy (TEM) analysis of HeLa cells transduced with proviral plasmid together with either HA-BCA2 or a control vector. In control cells, nascent assembled virions were observed on the plasma membrane and relatively little accumulation of virions was observed in intracellular compartments (Fig. 3A). In BCA2-expressing cells, however, substantial numbers of mature virions could be observed in the intracellular vesicles, and a significant reduction of mature viral particles on the plasma membrane was found (Fig. 3B). This suggests that BCA2 facilitates the internalization of mature viral particles into intracellular vesicles for degradation.

### BCA2 enhances the targeting of HIV-1 virions for lysosomal degradation

**Figure 4:**
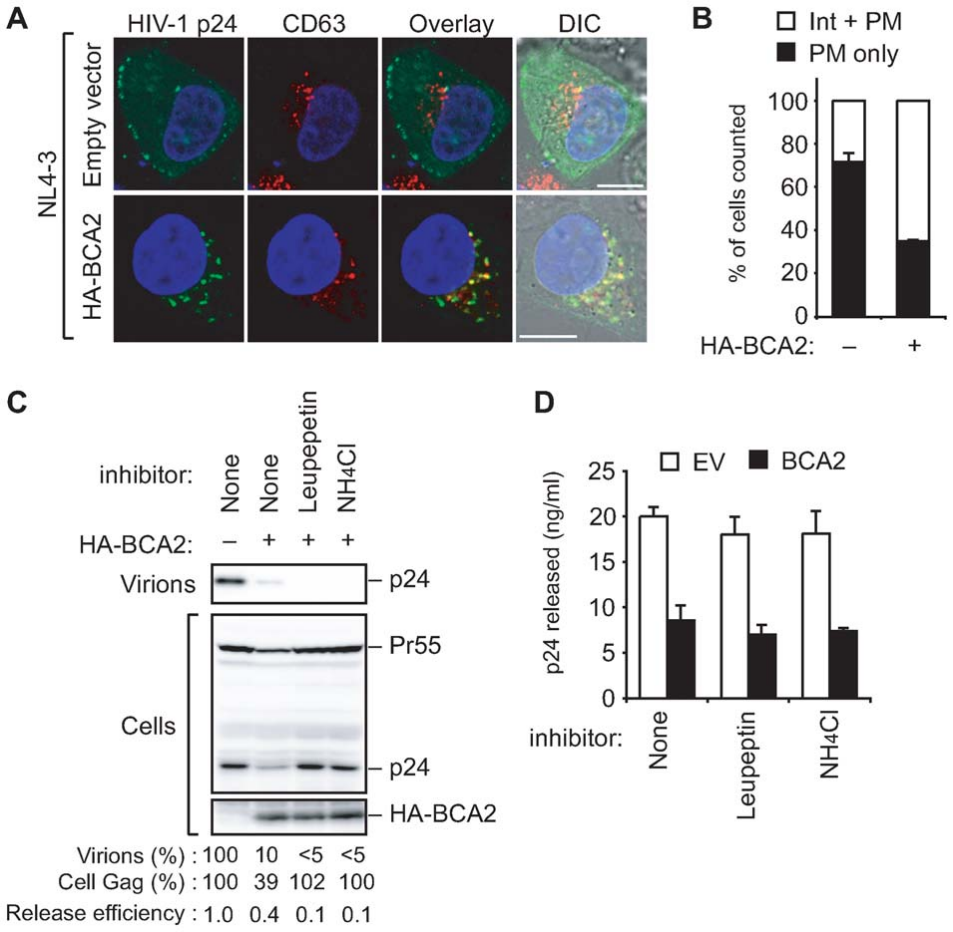
BCA2 enhances HIV-1 virion trafficking to lysosomes. (A) Confocal microscopic analysis of HeLa cells expressing pNL4–3 and either empty vector (top row) or pCMV-HA-BCA2 (bottom row), at a molar ratio of 1∶3 (scale bar, 10 µm). Note that these transfected cells also expressed Vpu. Cells were stained with anti-p24 (green) and anti-CD63 (red) antibodies and analyzed by confocal microscopy. (B) In the cultures described in (A), over 100 cells were analyzed for the subcellular localization of p24, which was either strongly evident at the plasma membrane (PM only), or intracellular accumulations as well as at the plasma membrane (Int + PM). The data are given as a percentage of the total cells. (C, D) HeLa cells transfected with 300 ng of pNL4–3 and either empty vector or pCMV-HA-BCA2, at a molar ratio of 1∶3, were treated with or without lysosomal inhibitors. Inhibitors were added to the medium 18 hours before harvesting. Cell lysates and supernatants were then analyzed by immunoblotting (C) and p24 ELISA (D). The final concentrations of leupeptin and NH_4_Cl were 5 µg/ml and 2 mM, respectively. The Gag signal intensities and the virus release efficiency are shown below the blots, as in Fig. 2F.

Consistent with our TEM results, immunofluorescent and confocal microscopic analysis further revealed that BCA2 expression promotes the accumulation of p24 in CD63^+^ intracellular compartments when compared with the vector control (Figs. 4A, B). Various proteins that are sorted into CD63^+^ intracellular compartments are destined for lysosomal degradation [30],[31]. To address whether virion degradation is mediated by this pathway following internalization, we co-transfected HeLa cells with the HIV-1 proviral plasmid together with either empty vector or HA-BCA2, and then treated the cells with lysosome inhibitors (leupeptin and NH_4_Cl). Strikingly, treatment with lysosome inhibitors significantly blocked the decrease in intracellular Gag in BCA2-expressing cells (Fig. 4C). Importantly also, parallel ELISA analysis of the supernatants from these transduced cells revealed that lysosome inhibitors had no effect upon viral release (Fig. 4D). These results suggest that BCA2 promotes the lysosomal degradation of HIV-1 virions following their retention on the plasma membrane and subsequent internalization into CD63^+^ endosomes.

### The targeted depletion of BCA2 reduces the intracellular accumulation of HIV-1 particles

**Figure 5:**
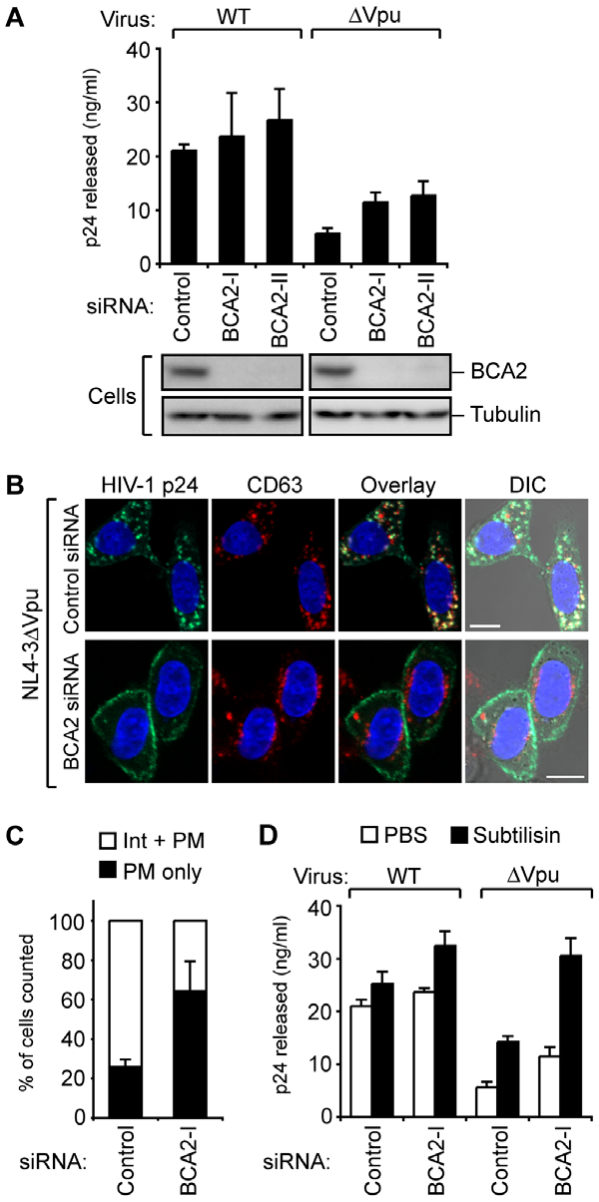
The targeted depletion of BCA2 blocks the intracellular accumulation of viral particles. (A) Single-round virus release analysis of HeLa cells treated with control siRNA or two different BCA2-targeted siRNA vectors for 24 hours, prior to transfection with pNL4–3 or pNL4–3ΔVpu. At 48 hours following transfection, cell supernatants were analyzed by p24 ELISA. Immunoblotting analysis with a BCA2 antibody to detect endogenous BCA2 expression is shown in the bottom panel. (B) Confocal microscopic analysis of HeLa cells treated with control siRNA (upper panels) or BCA2-targeted siRNA (lower panels), prior to transfection with pNL4–3ΔVpu (scale bar, 10 µm). After 48 hours following transfection, cells were fixed and immunostained with anti-p24 (green) and anti-CD63 (red) antibodies followed by confocal microscopy. (C) In the cultures described in (B), over 100 cells were analyzed for the subcellular localization of p24, as described in Fig. 4B. (D) HeLa cells were treated with control siRNA or BCA2-targeted siRNA for 24 hours, prior to transfection with pNL4–3 or pNL4–3ΔVpu as in (A). At 48 hours following transfection, cell supernatants were harvested (first supernatants) and cells were treated with either PBS or buffer containing the protease subtilisin (1 mg/ml) for 15 min, prior to re-harvesting of the cell supernatants (second supernatants). Both the first and second supernatants were then mixed and analyzed by p24 ELISA.

To further delineate the role of endogenous BCA2 in HIV-1 particle release, we next performed experiments in which HeLa cells were transduced with either control or two different BCA2-specific siRNAs (BCA2-I, II) and then transfected with pNL4-3 or pNL4-3ΔVpu. Immunoblotting analysis with a BCA2 antibody demonstrated that both of the siRNAs targeting BCA2 could significantly reduce its endogenous expression (Fig. 5A). Measurement of the p24 antigen levels in the cell supernatant further revealed that viral particle production was only slightly increased in both pNL4–3 and pNL4–3ΔVpu transfected cells, although the effect was more significant in pNL4–3ΔVpu transfected cells (approximately 2-fold) (Fig. 5A).

Immunofluorescent analysis by confocal microscopy additionally revealed that although the localization of Gag proteins was observed predominantly in CD63^+^ intracellular structures in control-siRNA treated cells, this profile was dramatically shifted to the plasma membrane in BCA2-siRNA treated cells (Figs. 5B, C). This indicated that the silencing of BCA2 blocks the relocation of virions into the intracellular compartments and increases the persistence of virions captured by tetherin on the cell surface.

To further investigate this possibility, siRNA-transduced cells were subsequently treated with the protease subtilisin, which liberates cell surface-captured virions by abolishing virion-tetherin interactions [15]. In the case of WT-virus, subtilisin stripping had only slight effects upon virion release (Fig. 5D), in agreement with a previous report [15]. However, in the case of Vpu-defective virus, viral release from BCA2-deplation cells was significantly recovered by subtilisin stripping, reaching the level of WT-virus infected cells (Fig. 5D). These data suggest that BCA2 depletion inhibits the intracellular accumulation of Gag proteins and, consequently, increases the fraction of virions retained at the cell surface by tetherin.

Overall, the results of our current study indicate that BCA2 facilitates the internalization of HIV-1 virions that have not been released, thereby enhancing their degradation. This internalization and degradation of cell surface-retained virions may represent rate limiting steps in the tetherin-mediated restriction of viral release that are accelerated by BCA2.

## Discussion

In our current study, we identify BCA2 as a functional tetherin-interacting protein. Although BCA2 is widely expressed in various cell lines [26],[32], its antiviral effects have been observed only in cells expressing tetherin, suggesting that BCA2 cooperates with tetherin to achieve efficient restriction of viral particle production. BCA2 was found in our current analyses to play a crucial role in the internalization and degradation of nascent HIV-1 virions, following their tethering to the host cell plasma membrane. These internalization and degradation steps may be rate limiting during restriction by tetherin because the targeted depletion of BCA2 can shift the distribution of Gag to the plasma membrane and can partly overcome the release inhibition of Vpu-minus virions in HeLa cells.

Importantly in this regard, BCA2 directs HIV-1 particles to CD63^+^ endosomes or lysosomes for degradation. The molecular mechanisms by which this is achieved have not yet been fully characterized. However, previous studies have demonstrated that BCA2 directly binds a small G protein, Rab7, and thereby plays crucial roles in vesicle trafficking to the late endosomes or lysosomes, in addition to lysosome biogenesis [26],[33]. Indeed, the aberrant expression of BCA2 not only affects epidermal growth factor receptor (EFGR) degradation, but also induces the perinuclear aggregation of lysosomes and increased acidity within lysosomes [26],[28]. Given our current data, these results indicate that BCA2 coordinates the trafficking of intracellular vesicles containing internalized viral particles to the lysosomes in conjunction with Rab7, resulting in the effective degradation of these virions. Consistently, in tetherin-positive HeLa cells, Gag protein has been shown to co-localize with the GTP-bound active form of Rab7 in the absence of Vpu (our unpublished observation). Furthermore, a dominant negative mutant of Rab5 can inhibit the internalization of nascent HIV-1 particles [15]. These findings raise the possibility that plasma membrane-tethered virions may go through a Rab5- and/or Rab7-dependent endocytotic pathway from the cell surface to the endosomes or lysosomes and eventual degradation. Notably, our immunoprecipitation data indicate that BCA2 interacts with tetherin at a region distinct from the Rab7 binding site. Consistently, an N-terminal truncation mutant of BCA2 can still interact with tetherin. These results suggest that BCA2 may simultaneously interact with Rab7 and tetherin at distinct regions and might therefore act as a physical scaffolding protein between these two proteins. During endocytosis, tetherin-BCA2 complexes might therefore recruit Rab7 to vesicles containing virions.

Although the function of BCA2 during HIV-1 restriction is likely to be dependent on tetherin, the antiviral effects of BCA2 were found to be still active against Vpu-positive viruses. However, the overexpression of Vpu can abrogate the antiviral effects of BCA2, indicating a potentially stoichiometric relationship between BCA2 and Vpu during BCA2-mediated viral restriction. Importantly, our current results suggest that BCA2 is not involved in regulating the expression of Vpu. However, it is possible that BCA2 antagonizes the function of Vpu in counteracting tetherin, although further analysis is needed to address this question.

Our current results additionally demonstrate that the effects of BCA2 depletion on particle production are about two-fold, which is a relatively modest impact compared with the 5-to-10-fold effects of Vpu in tetherin-positive cells. This indicates that the inhibition of BCA2 cannot fully restore Vpu-defective HIV-1 particle production to the level of the WT-virus. Apparently, capture of virions on the plasma membrane by tetherin provides restriction even when BCA2-depletion suppresses the internalization and degradation of nascent virions. These effected were further revealed by our subtilisin stripping assay; BCA2-depletion plus subtilisin treatment recovered ΔVpu-virus particle production to the level of the WT-virus. These results indicate that BCA2 very likely functions downstream of virus tethering on the plasma membrane (i.e. post-tethering stages).

In summary, the results of our current study demonstrate that BCA2 is a potential anti-HIV-1 host factor that partners with tetherin to facilitate the internalization and degradation of nascent viral particles. Our present findings thus shed new light on the molecular machinery underlying the tetherin-dependent HIV-1 restriction pathway. BCA2 and other molecules of this pathway may thus be potential new therapeutic targets for AIDS and its related disorders.

## Materials and Methods

### Cells and transfections

HeLa, HOS and 293T cells were cultured in DMEM supplemented with 10% fetal bovine serum (FBS). Jurkat cells were maintained in RPMI-1640 containing 10% FBS. Plasmid transfections into adherent or suspended cells were performed using Lipofectamine 2000 (Invitrogen, Carlsbad, CA) or Amaxa nucleofector (Program S-18; Amaxa biosystems, Cologne, Germany), respectively, according to the manufacturer's instructions.

### Plasmids and viruses

Human BCA2 and tetherin/CD317 coding sequences were amplified from HeLa total RNA by RT-PCR using the following pairs of oligonucleotides containing restriction enzyme BamHI sites (underlined) or a stop codon (boldface): 5′-GGATCCGGATGGCGGAGGCTTCGGCGGC-3′ (BCA2 forward primer, sense) and 5′-**TCA**GAAAGTCCATCGGTCATG-3′ (BCA2 reverse primer, antisense); 5′-GGATCCGGATGGCATCTACTTCGTATGA-3′ (tetherin forward primer, sense) and 5′-**TCA**CTGCAGCAGAGCGCTGAGGC-3′ (tetherin reverse primer, antisense). The purified PCR products were inserted into the pCR4Blunt-TOPO vector (Invitrogen), and cDNA inserts were then subcloned into pCMV-HA, pCMV-Myc, pEGFP-C1, pIRESpuro (Clontech, Palo Alto, CA) or pGEX-KG (Amersham Bioscience, Sunnyvale, CA) vectors. A human codon-optimized HIV-1 Vpu expression vector (pcDNA-Vphu) [34] and Vpu-deleted HIV-1 molecular clone (pNL4–3/Udel, herein called pNL4–3ΔVpu) [14] were kindly provided by Dr. K. Strebel (National Institutes of Health, Bethesda, MD). The ΔRING (1–227 aa), ΔN (148–305 aa), ΔC (1–147 aa), and C228A/C231A derivatives of BCA2, the tetherin mutant Δ1–20 (21–180 aa) and the truncated Vpu mutant (1–50 aa) were constructed using standard molecular cloning procedures. The WT-virus or ΔVpu-virus stocks were produced by transient transfection of 293T cells with the pNL4–3 or pNL4–3ΔVpu proviral plasmids, respectively. Culture supernatants containing virus were collected 48 hours after transfection, filtered through a 0.45 µm Millex-HV filter (Millipore, Billerica, MA) and immediately stored at −80°C until use.

### Antibodies

An anti-BCA2 polyclonal antibody was produced by UNITECH (Chiba, Japan). An anti-p24 monoclonal antibody has been described previously [35]. The rabbit anti-Vpu and mouse anti-HM1.24 (tetherin) antibodies were kindly donated by Dr. K. Strebel (National Institutes of Health, Bethesda, MD) [36] and Chugai Pharmaceutical Co. (Kanagawa, Japan) [37], respectively. Other antibodies used in this study were as follows: mouse anti-HA (Roche, Basel, Switzerland), mouse anti-Myc (Roche), mouse anti-α-tubulin (Sigma, St. Louis, MO), rabbit anti-CD63 (Santa Cruz Biotechnology, Santa Cruz, CA) and Alexafluor-conjugated anti-IgG (Invitrogen).

### In vitro binding assays

For GST pull-down assays, GST-tagged BCA2 was expressed in *Escherichia coli* BL21 (DE3) cells and purified using standard protocols. Myc-tetherin-expressing 293T cell lysates were incubated with glutathione-beads that had been coupled with GST-BCA2 proteins. The beads were then washed, and bound proteins were visualized by Coomassie Brilliant Blue R-250 staining and analyzed by immunoblotting. For immunoprecipitation analysis, 293T cells expressing Myc-tetherin and HA-BCA2 were lysed and incubated with an anti-HA affinity gel (Sigma). Alternatively, to detect endogenous tetherin-BCA2 complexes, HeLa cell lysates were co-incubated with protein A/G-mixed Sepharose (GE Healthcare, UK) and either anti-tetherin antibody or control mouse IgG. Bound proteins were analyzed by SDS-PAGE and immunoblotting.

### Single-round viral release and multi-cycle replication assays

Cells in 12-well plates were co-transfected with pNL4–3 or pNL4–3ΔVpu (300 ng) and either pCMV-HA-BCA2 or empty vector (0–300 ng), in the presence or absence of vectors encoding Vpu (30 or 75 ng) or Myc-tetherin (100 ng). Two days after transfection, virus-containing supernatants were harvested and filtrated to remove debris, and p24 antigens were measured by Lumipulse (Fujirebio, Tokyo, Japan). For immunoblotting assays, the virus-containing supernatants (400 µl) was layered onto 600 µl of 20% sucrose in PBS and centrifuged at 20,000 *g* for 2 hours at 4°C. The cell lysates were prepared using RIPA buffer by incubation at 4°C for 10 minutes and centrifugation at 16,000 *g* for 30 minutes. In experiments using lysosomal inhibitors, each drug was added 18 hours before harvesting. Immunoblotting band intensities were quantitated with ImageJ software.

For multi-cycle replication assays, Jurkat cells (1×10^6^) were transfected with either empty vector or pIRESpuro-BCA2 (3 µg). After the selection of transfectants with puromycin for 24 hours, cell aliquots were then infected with either HIV-1_NL4–3_ or HIV-1_NL4–3_Δ_Vpu_ at an m.o.i of 0.05. Viral supernatants were collected periodically, and p24 levels were measured as described above.

### Microscopy

One day prior to transfection, HeLa cells were seeded onto glass-bottom dishes coated with poly-L-lysine (Matsunami, Osaka, Japan). At 48 hours after transfection, the cells were fixed with 4% paraformaldehyde and permeabilized with 1% Triton X-100. Cells were than stained with primary antibodies and Alexa-conjugated secondary antibodies. Confocal microscopic imaging was performed using a Zeiss LSM510 instrument equipped with a 63× oil-immersion objective. For electron microscopy, transfected HeLa cells were fixed with 2.5% glutaraldehyde and subjected to transmission electron microscopy, as described previously [38].

### Pulse-chase radiolabeling

Cells in 6-well plates were co-transfected with pNL4–3ΔVpu (1 µg) and either pCMV-HA-BCA2 or empty vector (3 µg). Two days after transfection, the cells were washed and starved in Met-/Cys-depletion medium (Invitrogen) for 30 min and pulse-labeled for 15 min with 0.25 mCi/ml of [^35^S]Met-Cys medium, and chased in unlabeled medium for 4.5 hours. Cells were harvested periodically, and cell lysates were immunoprecipitated with anti-p24 antibody, and then analyzed by SDS-PAGE and autoradiography.

### siRNA knockdown and subtilisin stripping assays

BCA2-targeted siRNAs were obtained from Invitrogen as Stealth Select RNAi constructs (Oligo ID #HSS120532 and #HSS120534). A Stealth RNAi Luciferase reporter control (Invitrogen) was used as the negative control siRNA. Cells in 12-well plates were transfected with these siRNAs at a final concentration of 50 µM using Lipofectamine RNAiMAX (Invitrogen). The following day, the cells were re-transfected with 300 ng of either pNL4–3 or pNL4–3ΔVpu, and two days later were harvested and analyzed by immunoblotting or confocal microscopy.

For protease subtilisin stripping assays, viral supernatants (1.2 ml) from siRNA/DNA-transfected HeLa cells were harvested as a “first supernatant”. After harvesting, the cells were washed once with pre-warmed PBS and then incubated with 300 µl of either PBS or Tris/HCl (pH 8.0) buffer containing 1 mg/ml subtilisin (Sigma) for 15 min at 37°C. To stop the reaction, 900 µl of DMEM containing 5 mM PMSF were added to the cells, and supernatants (total 1.2 ml) were again harvested as a “second supernatant”. Both the first and second supernatants were then mixed and the p24 levels were measured as described above.

### Accession numbers

The GenBank accession numbers for human BCA2 (Rabring7/ZNF364/RNF115) and human tetherin (CD317/BST-2/HM1.24) are BC054049 and D28137, respectively.

## Supporting Information

Figure S1 Endogenous expression of tetherin and BCA2 in the cells used in this study. (A) Flow cytometric analysis of cell surface tetherin expression in HeLa, HOS and Jurkat cells. Cells were washed with ice-cold PBS containing 1% BSA, and were blocked for 10 min with 10% normal goat serum. The cells were then stained with an anti-tetherin monoclonal antibody (0.1 µg/ml) and a PE-conjugated secondary antibody (Beckman Coulter, Fullerton, CA). All samples were analyzed with a FACS Caliber (BD Biosciences, San Jose, CA). (B) Immunoblotting analysis of the indicated cell lysates. Blots were probed with either anti-BCA2 or anti-α-tubulin antibodies.
(0.20 MB PDF)

Figure S2 BCA2 has no detectable effects on the Gag RNA levels. RT-PCR analysis of total RNA extracted from HeLa cells transfected with pNL4–3 and either control vector or pCMV-HA-BCA2 at a molar ratio of 1∶1. The PCR primers were as follows: 5′-CCCTATAGTGCAGAACCTCCA-3′ (p24CA RT-forward) and 5′-CATTATGGTAGCTGGATTTGTTAC-3′ (p24CA RT-reverse); 5′-GATCCGGTACTAGAGGAACTGAAAAAC-3′ (Exogenous BCA2 RT-forward) and 5′-TCACTGCAGCAGAGCGCTGAGGC-3′ (Exogenous BCA2 RT-reverse); 5′-ACGGATGGACTTTCTGAAGC-3′ (Endogenous BCA2 RT-forward) and 5′-AAGGCAACATGACAGACAGC-3′ (Endogenous BCA2 RT-reverse). The G3PDH RT-primers used have been described previously [35]. To clarify the differences between exogenous and endogenous BCA2, the exogenous BCA2 RT-forward primer contains a vector-derived sequence. Numerical values below the blots indicate the Gag signal intensities normalized to the G3PDH values determined by densitometry.
(0.08 MB PDF)

## References

[1] Goff SP (2007). Host factors exploited by retroviruses.. Nat Rev Microbiol.

[2] Huthoff H, Towers GJ (2008). Restriction of retroviral replication by APOBEC3G/F and TRIM5alpha.. Trends Microbiol.

[3] Freed EO (2004). HIV-1 and the host cell: an intimate association.. Trends Microbiol.

[4] Malim MH, Emerman M (2008). HIV-1 accessory proteins–ensuring viral survival in a hostile environment.. Cell Host Microbe.

[5] Neil SJ, Sandrin V, Sundquist WI, Bieniasz PD (2007). An interferon-alpha-induced tethering mechanism inhibits HIV-1 and Ebola virus particle release but is counteracted by the HIV-1 Vpu protein.. Cell Host Microbe.

[6] Neil SJ, Zang T, Bieniasz PD (2008). Tetherin inhibits retrovirus release and is antagonized by HIV-1 Vpu.. Nature.

[7] Van Damme N, Goff D, Katsura C, Jorgenson RL, Mitchell R (2008). The interferon-induced protein BST-2 restricts HIV-1 release and is downregulated from the cell surface by the viral Vpu protein.. Cell Host Microbe.

[8] Jouvenet N, Neil SJ, Zhadina M, Zang T, Kratovac Z (2009). Broad-spectrum inhibition of retroviral and filoviral particle release by tetherin.. J Virol.

[9] Sakuma T, Noda T, Urata S, Kawaoka Y, Yasuda J (2009). Inhibition of Lassa and Marburg virus production by tetherin.. J Virol.

[10] Kaletsky RL, Francica JR, Agrawal-Gamse C, Bates P (2009). Tetherin-mediated restriction of filovirus budding is antagonized by the Ebola glycoprotein.. Proc Natl Acad Sci U S A.

[11] Strebel K, Klimkait T, Martin MA (1988). A novel gene of HIV-1, vpu, and its 16-kilodalton product.. Science.

[12] Cohen EA, Terwilliger EF, Sodroski JG, Haseltine WA (1988). Identification of a protein encoded by the vpu gene of HIV-1.. Nature.

[13] Huet T, Cheynier R, Meyerhans A, Roelants G, Wain-Hobson S (1990). Genetic organization of a chimpanzee lentivirus related to HIV-1.. Nature.

[14] Klimkait T, Strebel K, Hoggan MD, Martin MA, Orenstein JM (1990). The human immunodeficiency virus type 1-specific protein vpu is required for efficient virus maturation and release.. J Virol.

[15] Neil SJ, Eastman SW, Jouvenet N, Bieniasz PD (2006). HIV-1 Vpu promotes release and prevents endocytosis of nascent retrovirus particles from the plasma membrane.. PLoS Pathog.

[16] Willey RL, Maldarelli F, Martin MA, Strebel K (1992). Human immunodeficiency virus type 1 Vpu protein induces rapid degradation of CD4.. J Virol.

[17] Willey RL, Maldarelli F, Martin MA, Strebel K (1992). Human immunodeficiency virus type 1 Vpu protein regulates the formation of intracellular gp160-CD4 complexes.. J Virol.

[18] Chen MY, Maldarelli F, Karczewski MK, Willey RL, Strebel K (1993). Human immunodeficiency virus type 1 Vpu protein induces degradation of CD4 in vitro: the cytoplasmic domain of CD4 contributes to Vpu sensitivity.. J Virol.

[19] Miyagi E, Andrew AJ, Kao S, Strebel K (2009). Vpu enhances HIV-1 virus release in the absence of Bst-2 cell surface down-modulation and intracellular depletion.. Proc Natl Acad Sci U S A.

[20] Gottlinger HG, Dorfman T, Cohen EA, Haseltine WA (1993). Vpu protein of human immunodeficiency virus type 1 enhances the release of capsids produced by gag gene constructs of widely divergent retroviruses.. Proc Natl Acad Sci U S A.

[21] Strebel K, Klimkait T, Maldarelli F, Martin MA (1989). Molecular and biochemical analyses of human immunodeficiency virus type 1 vpu protein.. J Virol.

[22] Terwilliger EF, Cohen EA, Lu YC, Sodroski JG, Haseltine WA (1989). Functional role of human immunodeficiency virus type 1 vpu.. Proc Natl Acad Sci U S A.

[23] Harila K, Prior I, Sjoberg M, Salminen A, Hinkula J (2006). Vpu and Tsg101 regulate intracellular targeting of the human immunodeficiency virus type 1 core protein precursor Pr55gag.. J Virol.

[24] Harila K, Salminen A, Prior I, Hinkula J, Suomalainen M (2007). The Vpu-regulated endocytosis of HIV-1 Gag is clathrin-independent.. Virology.

[25] Van Damme N, Guatelli J (2008). HIV-1 Vpu inhibits accumulation of the envelope glycoprotein within clathrin-coated, Gag-containing endosomes.. Cell Microbiol.

[26] Mizuno K, Kitamura A, Sasaki T (2003). Rabring7, a novel Rab7 target protein with a RING finger motif.. Mol Biol Cell.

[27] Adachi A, Gendelman HE, Koenig S, Folks T, Willey R (1986). Production of acquired immunodeficiency syndrome-associated retrovirus in human and nonhuman cells transfected with an infectious molecular clone.. J Virol.

[28] Sakane A, Hatakeyama S, Sasaki T (2007). Involvement of Rabring7 in EGF receptor degradation as an E3 ligase.. Biochem Biophys Res Commun.

[29] Amemiya Y, Azmi P, Seth A (2008). Autoubiquitination of BCA2 RING E3 ligase regulates its own stability and affects cell migration.. Mol Cancer Res.

[30] Luzio JP, Rous BA, Bright NA, Pryor PR, Mullock BM (2000). Lysosome-endosome fusion and lysosome biogenesis.. J Cell Sci.

[31] Piper RC, Luzio JP (2001). Late endosomes: sorting and partitioning in multivesicular bodies.. Traffic.

[32] Burger AM, Gao Y, Amemiya Y, Kahn HJ, Kitching R (2005). A novel RING-type ubiquitin ligase breast cancer-associated gene 2 correlates with outcome in invasive breast cancer.. Cancer Res.

[33] Burger A, Amemiya Y, Kitching R, Seth AK (2006). Novel RING E3 ubiquitin ligases in breast cancer.. Neoplasia.

[34] Nguyen KL, llano M, Akari H, Miyagi E, Poeschla EM (2004). Codon optimization of the HIV-1 vpu and vif genes stabilizes their mRNA and allows for highly efficient Rev-independent expression.. Virology.

[35] Ohba K, Ryo A, Dewan MZ, Nishi M, Naito T (2009). Follicular dendritic cells activate HIV-1 replication in monocytes/macrophages through a juxtacrine mechanism mediated by P-selectin glycoprotein ligand 1.. J Immunol.

[36] Maldarelli F, Chen MY, Willey RL, Strebel K (1993). Human immunodeficiency virus type 1 Vpu protein is an oligomeric type I integral membrane protein.. J Virol.

[37] Ozaki S, Kosaka M, Wakahara Y, Ozaki Y, Tsuchiya M (1999). Humanized anti-HM1.24 antibody mediates myeloma cell cytotoxicity that is enhanced by cytokine stimulation of effector cells.. Blood.

[38] Ryo A, Tsurutani N, Ohba K, Kimura R, Komano J (2008). SOCS1 is an inducible host factor during HIV-1 infection and regulates the intracellular trafficking and stability of HIV-1 Gag.. Proc Natl Acad Sci U S A.

