# Supplementary figures and images for: BCA2/Rabring7 Promotes Tetherin-Dependent HIV-1 Restriction
Source: PLoS Pathog. 2009 Dec 18;5(12):e1000700. doi: 10.1371/journal.ppat.1000700 (PMC2788703; doi:10.1371/journal.ppat.1000700)

**A**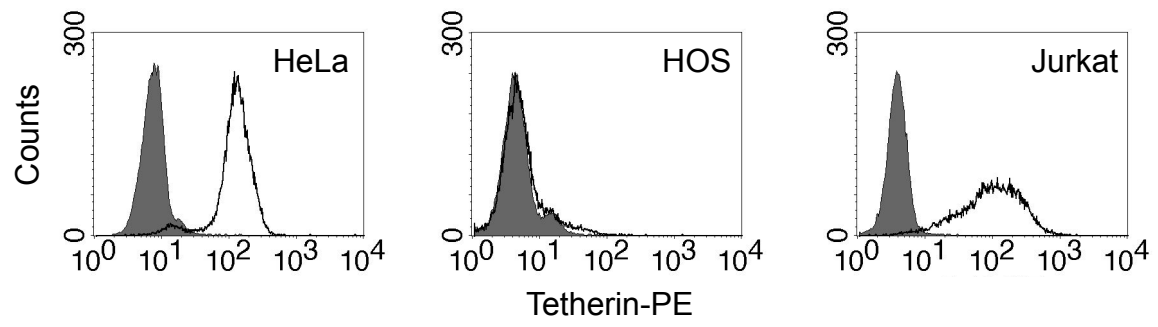**B**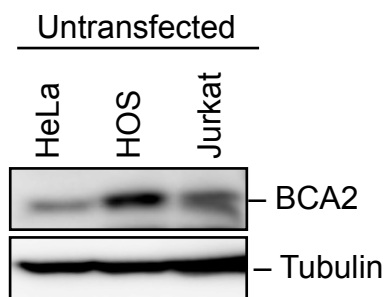

Supplement: Figure S1 — Endogenous expression of tetherin and BCA2 in the cells used in this study. (A) Flow cytometric analysis of cell surface tetherin expression in HeLa, HOS and Jurkat cells. Cells were washed with ice-cold PBS containing 1% BSA, and were blocked for 10 min with 10% normal goat serum. The cells were then stained with an anti-tetherin monoclonal antibody (0.1 µg/ml) and a PE-conjugated secondary antibody (Beckman Coulter, Fullerton, CA). All samples were analyzed with a FACS Caliber (BD Biosciences, San Jose, CA). (B) Immunoblotting analysis of the indicated cell lysates. Blots were probed with either anti-BCA2 or anti-α-tubulin antibodies. (0.20 MB PDF) [file ppat.1000700.s001.pdf]

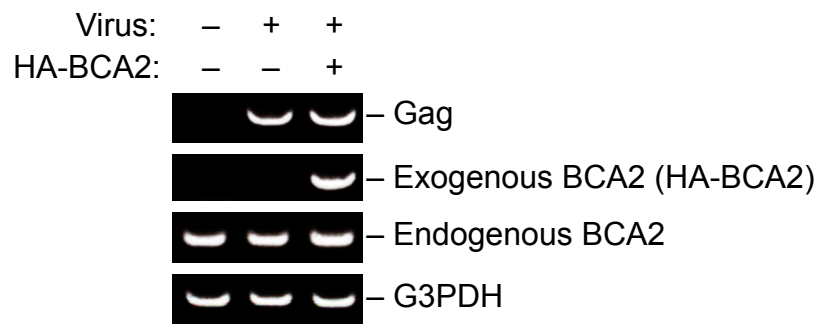

*Gag/G3PDH (%)*:    -    100    117

Supplement: Figure S2 — BCA2 has no detectable effects on the Gag RNA levels. RT-PCR analysis of total RNA extracted from HeLa cells transfected with pNL4–3 and either control vector or pCMV-HA-BCA2 at a molar ratio of 1∶1. The PCR primers were as follows: 5′-CCCTATAGTGCAGAACCTCCA-3′ (p24CA RT-forward) and 5′-CATTATGGTAGCTGGATTTGTTAC-3′ (p24CA RT-reverse); 5′-GATCCGGTACTAGAGGAACTGAAAAAC-3′ (Exogenous BCA2 RT-forward) and 5′-TCACTGCAGCAGAGCGCTGAGGC-3′ (Exogenous BCA2 RT-reverse); 5′-ACGGATGGACTTTCTGAAGC-3′ (Endogenous BCA2 RT-forward) and 5′-AAGGCAACATGACAGACAGC-3′ (Endogenous BCA2 RT-reverse). The G3PDH RT-primers used have been described previously [35]. To clarify the differences between exogenous and endogenous BCA2, the exogenous BCA2 RT-forward primer contains a vector-derived sequence. Numerical values below the blots indicate the Gag signal intensities normalized to the G3PDH values determined by densitometry. (0.08 MB PDF) [file ppat.1000700.s002.pdf]
